# A ROS-responsive nanoplatform for synergistic pain relief and gastrointestinal recovery in intestinal ischemia-reperfusion injury

**DOI:** 10.3389/fped.2026.1873783

**Published:** 2026-09-21

**Authors:** Yingying Jing, Gaofeng Xian, Gang Wu, Lingna Ye, Hanshu Wang, Xiaoni Cai

**Affiliations:** 1 Department of Nursing, Shangyu People's Hospital of Shaoxing, Shaoxing University, Shangyu, China; 2 Department of Gastrointestinal Surgery, Shangyu People's Hospital of Shaoxing, Shaoxing University, Shangyu, China; 3 Department of Critical Care Medicine, Shangyu People's Hospital of Shaoxing, Shaoxing University, Shangyu, China; 4 Department of General Surgery, Shangyu People's Hospital of Shaoxing, Shaoxing University, Shangyu, China

**Keywords:** analgesia, gastrointestinal functional recovery, intestinal ischemia-reperfusion, nanodelivery, ROS-responsive

## Abstract

**Introduction:**

There is a clinical contradiction between postoperative analgesia and gastrointestinal functional recovery following intestinal ischemia-reperfusion (I/R) injury.

**Methods:**

In this study, a reactive oxygen species (ROS)-responsive nanoplatform (LA-CLX-NP) was constructed by conjugating DSPE-PEG with α-lipoic acid (LA) followed by self-assembly and encapsulation of celecoxib.

**Results:**

The nanoparticles exhibited uniform size, good stability, and ROS/pH dual-responsive drug release properties. *In vitro*, LA-CLX-NP was efficiently internalized by macrophages and, compared with free drugs, more effectively scavenged ROS, restored redox balance, and modulated inflammatory cytokines. In a mouse model of intestinal I/R injury, LA-CLX-NP significantly reduced diarrhea incidence, increased mechanical withdrawal threshold, decreased abdominal licking and scratching behaviors, preserved ZO-1 tight junctions, suppressed COX-2 overexpression, and normalized serum cytokine profiles, with superior efficacy to single agents.

**Discussion:**

This strategy achieves on-demand delivery of celecoxib to the inflamed intestine, exerts synergistic anti-inflammatory and antioxidant effects, and thereby reconciles effective pain relief with rapid gastrointestinal recovery, offering a new approach for postoperative management of intestinal I/R injury.

## 1 Introduction

Intestinal ischemia-reperfusion (I/R) injury is a common and severe pathological condition encountered in various surgical procedures, including intestinal transplantation, mesenteric artery occlusion, and hemorrhagic shock (1, 2). This process is characterized by an initial ischemic insult followed by a robust inflammatory response upon reperfusion, leading to excessive production of reactive oxygen species (ROS), activation of inflammatory cascades, and disruption of the intestinal mucosal barrier (3, 4). Clinically, patients with intestinal I/R injury often suffer from severe abdominal pain, diarrhea, delayed gastrointestinal function recovery, and prolonged hospitalization (5, 6).

Postoperative pain management is critical for patient comfort and recovery, but it remains a clinical dilemma. Conventional systemic analgesic strategies, particularly opioids, effectively relieve pain but concurrently inhibit gastrointestinal motility, resulting in constipation, ileus, and delayed recovery (7, 8). Nonsteroidal anti-inflammatory drugs (NSAIDs) are often used as alternatives or adjuvants; however, their systemic administration is associated with dose-dependent adverse effects, including gastrointestinal irritation, renal impairment, and cardiovascular risks (9, 10). Selective cyclooxygenase-2 (COX-2) inhibitors (e.g., celecoxib) offer improved gastrointestinal safety profiles by sparing COX-1-mediated gastric mucosal protection (11). Nevertheless, systemic administration of celecoxib still suffers from poor water solubility, limited bioavailability, and potential cardiovascular and renal side effects (12, 13). More importantly, the fundamental contradiction between achieving effective analgesia and maintaining gastrointestinal function remains unresolved.

Nanotechnology-based drug delivery systems provide new opportunities to address these challenges (14, 15). In particular, stimuli-responsive nanocarriers enable spatiotemporally controlled drug release at pathological sites, thereby enhancing therapeutic efficacy while reducing systemic toxicity (16, 17). Given that intestinal I/R injury is characterized by massive ROS production in the injured tissue (18, 19), ROS-responsive nanocarriers offer a unique opportunity for targeted delivery. Among various ROS-responsive moieties, α-lipoic acid (LA) is a naturally occurring antioxidant whose unique disulfide five-membered ring structure is cleaved under oxidative conditions (20). Notably, LA itself possesses potent antioxidant and anti-inflammatory activities, making it an ideal building block for constructing “therapeutic carriers” that combine drug delivery with intrinsic pharmacological effects (21, 22).

Based on the above considerations, we designed a ROS-responsive nanoplatform self-assembled from LA-NP to encapsulate the hydrophobic drug celecoxib. This platform achieves synergistic therapy by integrating three functions (Scheme 1): the DSPE-PEG backbone confers long-circulating properties and enables passive targeting to inflamed intestinal tissue via the enhanced permeability and retention (EPR) effect; the LA moiety acts as a ROS-responsive “switch”, triggering nanoparticle disassembly and drug release in the high-ROS microenvironment of local inflammation, while simultaneously serving as an auxiliary therapeutic agent that directly scavenges ROS and suppresses inflammatory signaling pathways; the released celecoxib selectively inhibits COX-2-mediated prostaglandin synthesis, synergizing with the antioxidant effect of LA to break the vicious cycle of inflammation and oxidative injury. This study aimed to construct an LA-modified ROS-responsive nanoparticle loaded with celecoxib (LA-CLX-NP) and to validate its ability to preferentially accumulate in the injured intestinal tissue, release the drug upon local ROS stimulation, and exert synergistic anti-inflammatory and antioxidant effects, thereby protecting gastrointestinal function while achieving effective analgesia. To this end, we systematically characterized the physicochemical properties and ROS-responsive behavior of the nanoparticles, evaluated their cellular uptake and anti-inflammatory activity in RAW 264.7 macrophages, and assessed their *in vivo* therapeutic efficacy with a focus on pain-related behaviors, intestinal barrier integrity, and gastrointestinal functional recovery.

**Scheme 1:**
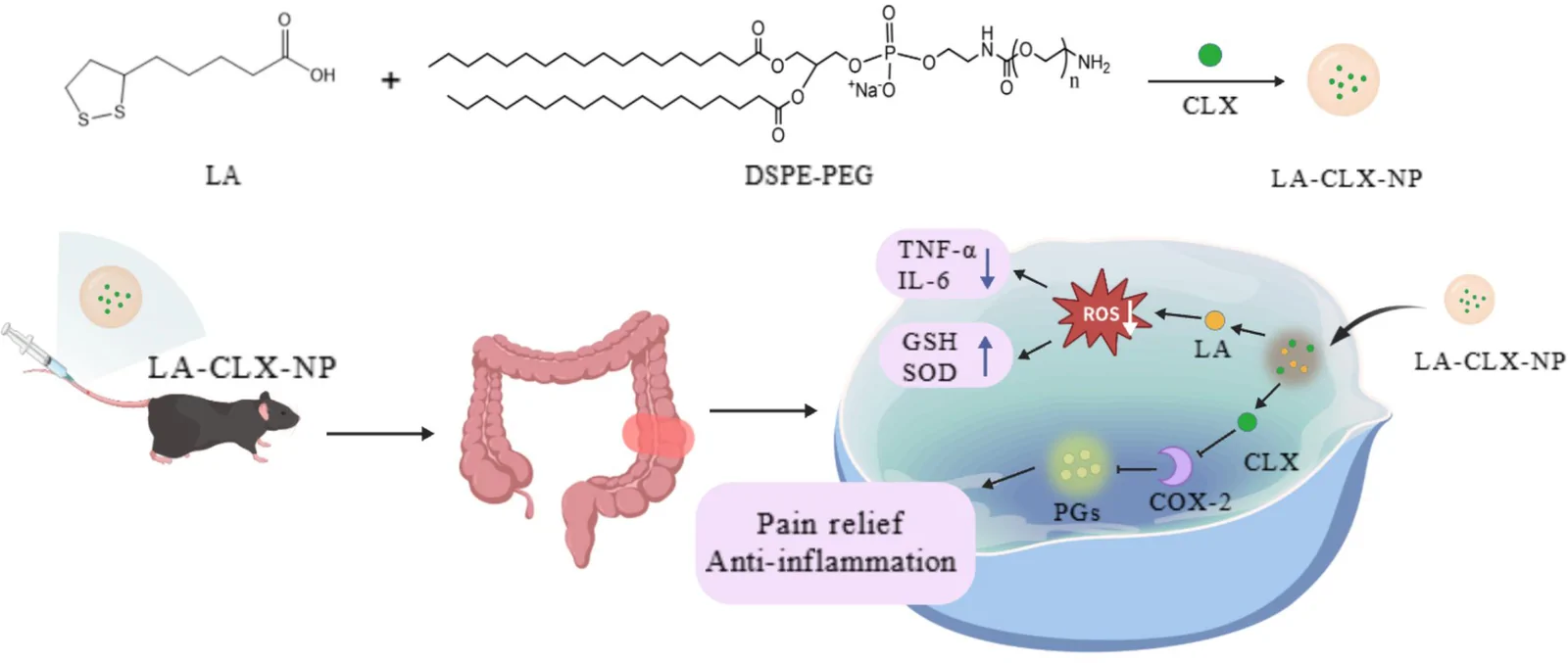
Schematic illustration of the ROS-responsive nanoplatform (LA-CLX-NP) for synergistic therapy of intestinal ischemia-reperfusion injury. Created with BioGDP.com (26).

Recent advances have demonstrated the promise of ROS-responsive nanoplatforms in this field. For instance, Sheng et al. (23) developed macrophage membrane-coated MnO_2_ nanoparticles loaded with rapamycin to alleviate intestinal I/R injury by scavenging ROS and promoting autophagy, relying on the enzyme-mimetic activity of MnO_2_ to decompose H_2_O_2_ into O_2_ and utilizing macrophage membrane coating for targeting. Yu et al. (24) reported oxidation-responsive PEG-poly(α-lipoic acid) nanoparticles for coenzyme Q10 delivery to attenuate hepatic I/R injury. In the context of chronic intestinal inflammation, Wang et al. (25) constructed ROS-responsive topology-switching nanogels capable of enzymatically degrading cfDNA, thereby blocking TLR9 signaling activation at the source. Compared with these systems, LA-CLX-NP offers distinct innovations: (i) material design—whereas previous studies have utilized LA as a copolymer backbone or auxiliary component, our system employs LA as both a ROS-responsive “switch” and a therapeutic entity, with the disulfide bond cleavage releasing LA molecules that directly exert antioxidant effects, embodying a “carrier-as-drug” concept; (ii) drug synergy—while existing platforms typically deliver a single class of anti-inflammatory or antioxidant agents, this study achieves, for the first time, the synergistic co-delivery of a COX-2 inhibitor (CLX) and an endogenous antioxidant (LA), targeting both “inflammation initiation” and “oxidative damage” to establish a complete “anti-inflammatory plus antioxidant” loop. This study provides a proof-of-concept for an intelligent targeted analgesic strategy that reconciles the clinical contradiction between effective pain control and rapid gastrointestinal rehabilitation, offering a new approach for the postoperative management of intestinal I/R injury.

## 2 Materials and methods

### 2.1 Materials

The RAW264.7 mouse monocyte/macrophage cell line was purchased from the American Type Culture Collection (ATCC, USA). SPF-grade male C57BL/6 mice (6–8 weeks old, body weight 20–25 g) were obtained from Beijing Weitong Lihua Experimental Animal Technology Co., Ltd. (Beijing, China) and all animal experiments were approved by the Animal Ethics Committee of Shangyu People's Hospital of Shaoxing (approval No. 2026042722). L-Arginine (L-Arg, purity ≥98%), nicotinamide mononucleotide (NMN, purity ≥95%), lipopolysaccharide (LPS, from Escherichia coli O111:B4) and fluorescein isothiocyanate (FITC) were purchased from Shanghai Aladdin Biochemical Technology Co., Ltd. Oxidized low-density lipoprotein (ox-LDL), soybean phospholipid, cholesterol, and 1,2-distearoyl-sn-glycero-3-phosphoethanolamine-polyethylene glycol 2,000 (DSPE-PEG2000) were purchased from Beijing Solarbio Science & Technology Co., Ltd. α-Lipoic acid (LA, purity ≥99%), 1-ethyl-3-(3-dimethylaminopropyl)carbodiimide hydrochloride (EDC·HCl), N-hydroxysuccinimide (NHS), and triethylamine (TEA) were purchased from Shanghai Macklin Biochemical Technology Co., Ltd. Celecoxib (purity ≥98%) was purchased from Dalian Meilun Biotechnology Co., Ltd. Chloroform, methanol, and dimethyl sulfoxide (DMSO) were of analytical grade and purchased from Sinopharm Chemical Reagent Co., Ltd. Fetal bovine serum (FBS), DMEM high-glucose medium (with L-glutamine), and penicillin-streptomycin (100×) were purchased from Gibco. Trypsin-EDTA (0.25%) and phosphate-buffered saline (PBS, pH 7.4) were purchased from Beijing Solarbio Science & Technology Co., Ltd. Cell culture flasks, plates, and confocal dishes were purchased from Corning. The CCK-8 cell viability assay kit, Mouse PGE2 ELISA kit, reactive oxygen species assay kit (DCFH-DA), Near-infrared fluorescent dye DiR and total nitric oxide assay kit (Griess method) were purchased from Beyotime Biotechnology (Shanghai, China). Mouse IL-6, TNF-α, and IL-10 ELISA kits were purchased from Wuhan Servicebio Technology Co., Ltd. Reduced glutathione (GSH), oxidized glutathione (GSSG), and superoxide dismutase (SOD) activity assay kits were purchased from Nanjing Jiancheng Bioengineering Institute. Malondialdehyde (MDA) assay kit was also purchased from Nanjing Jiancheng Bioengineering Institute. Rabbit anti-mouse COX-2 monoclonal antibody, rabbit anti-mouse ZO-1 monoclonal antibody, and rabbit anti-mouse β-actin monoclonal antibody were purchased from Abcam. Horseradish peroxidase (HRP)-conjugated goat anti-rabbit secondary antibody was purchased from Beijing Zhongshan Jinqiao Biotechnology Co., Ltd. Primers for COX-2 and GAPDH were synthesized by Sangon Biotech (Shanghai) Co., Ltd.

### 2.2 Methods

#### 2.2.1 Synthesis of LA-NP

LA-NP was synthesized via an amidation reaction. Briefly, α-lipoic acid (LA, 5.55 mg, 26.9 µmol) was dissolved in anhydrous DMSO, followed by sequential addition of EDC·HCl (10.35 mg, 54 µmol) and NHS (6.2 mg, 54 µmol). The mixture was activated at room temperature for 30 min. Then DSPE-PEG_2000_-NH_2_ (50 mg, 18 µmol) and TEA (10 µL) were added, and the reaction was stirred under nitrogen protection at room temperature in the dark for 24 h. The reaction mixture was transferred to a dialysis bag (MWCO 3,500 Da) and dialyzed against ultrapure water for 48 h, with water changed every 12 h. The dialysate was lyophilized to obtain a light yellow solid product (LA-NP), which was stored at −20 °C in the dark until use.

#### 2.2.2 Structural characterization

The structures of LA, CLX, and LA-NP were confirmed by proton nuclear magnetic resonance (^1^H NMR) spectroscopy and Fourier-transform infrared (FT-IR) spectroscopy. FT-IR spectra were recorded using the KBr pellet method in the range of 4,000–400 cm^−1^. UV-Vis absorption spectra were recorded by full-wavelength scanning from 200 to 800 nm.

#### 2.2.3 Preparation of nanoparticles

Celecoxib-loaded nanoparticles were prepared by the thin-film hydration method. LA-NP (5 mg) and celecoxib (0.25 mg) were accurately weighed and dissolved in a chloroform-methanol mixture (3:1, v/v, 5 mL). The solution was transferred to a round-bottom flask and the organic solvent was removed by rotary evaporation at 40 °C, forming a uniform film on the flask wall. The film was further dried under vacuum overnight to completely remove residual solvent. The film was hydrated with PBS (8 mL, pH 7.4) for 1 h with gentle shaking. The hydrated dispersion was then placed in an ice bath and sonicated using a probe sonicator to homogenize the particle size. Finally, the dispersion was filtered through a 0.22 µm aqueous membrane to obtain the drug-loaded nanoparticles, designated as LA-CLX-NP. Blank nanoparticles (LA-NP, without celecoxib) were prepared using the same method.

#### 2.2.4 Physicochemical characterization of nanoparticles

Particle size, polydispersity index (PDI), and zeta potential were measured by dynamic light scattering (DLS) with three replicates per sample. Morphology was observed by transmission electron microscopy (TEM): a drop of nanoparticle suspension was placed on a copper grid and allowed to air-dry before imaging.

#### 2.2.5 Determination of drug loading and encapsulation efficiency

The content of celecoxib was determined by high-performance liquid chromatography (HPLC). Chromatographic conditions: C18 column (4.6 mm × 250 mm, 5 µm); mobile phase acetonitrile-water (65:35, v/v); flow rate 1.0 mL/min; detection wavelength 254 nm; column temperature 30 °C. An aliquot of the nanoparticle solution was centrifuged at 14,000 rpm for 30 min, and the supernatant was filtered through a 0.22 µm filter before injection. The drug loading (DL%) and encapsulation efficiency (EE%) were calculated as follows (Supplementary Table S1):

$$\mathrm{EE}(\text{\%})=\frac{\left(\mathrm{amount}\;\mathrm{of}\;\mathrm{drug}\;\mathrm{added}-\mathrm{free}\;\mathrm{drug}\right)}{\mathrm{amount}\;\mathrm{of}\;\mathrm{drug}\;\mathrm{added}\;}\;\times 100\text{\%}$$

$$\mathrm{DL}(\text{\%})=\frac{\left(\mathrm{amount}\;\mathrm{of}\;\mathrm{drug}\;\mathrm{added}\;-\mathrm{free}\;\mathrm{drug}\right)}{\mathrm{total}\;\mathrm{nanoparticle}\;\mathrm{weight}}\;\times 100\text{\%}$$

#### 2.2.6 *In vitro* ROS-responsive release

The ROS-responsive drug release behavior was investigated using a dialysis bag method. LA-CLX-NP and LA-NP (each containing an equivalent dose of celecoxib) were placed into dialysis bags (MWCO 3,500 Da). The bags were sealed and immersed in PBS release medium (containing 0.5% Tween 80) with different pH (6.8, 7.4) and different H_2_O_2_ concentrations (0.1, 0.25, or 5 mmol/L). The samples were incubated at 37 °C with shaking at 100 r/min in the dark. At predetermined time points (1, 2, 4, 6, 8, 12, 24 h), 1 mL of the release medium was withdrawn and replaced with an equal volume of fresh medium. The samples were filtered through a 0.22 µm filter, and the concentrations of celecoxib and α-lipoic acid were determined by HPLC and UV spectrophotometry, respectively. Cumulative release rates were calculated.

Specifically, to evaluate the release behavior under physiologically relevant oxidative stress conditions, LA-CLX-NP was incubated in PBS (pH 7.4) containing 250 µM H_2_O_2_. The cumulative releases of CLX and LA at 24 h were determined to be 41.94% and 38.97%, respectively, demonstrating a concentration-dependent release profile when compared with the lower (0.1 mM) and higher (5 mM) H_2_O_2_ conditions.

#### 2.2.7 Synthesis of non-responsive CLX-loaded nanoparticles (CLX-NP)

As a non-ROS-responsive control, CLX-loaded nanoparticles without LA modification (CLX-NP) were prepared using the same thin-film hydration method. Briefly, DSPE-PEG-OMe (5 mg) and celecoxib (0.25 mg, mass ratio 20:1) were dissolved in chloroform-methanol (3:1, v/v, 5 mL). The organic solvent was removed by rotary evaporation at 40 °C to form a uniform film, which was further dried under vacuum overnight. The film was hydrated with PBS (8 mL, pH 7.4) for 1 h with gentle shaking, followed by probe sonication in an ice bath (100 W, 2 s on/2 s off, total 6 min). The dispersion was filtered through a 0.22 µm membrane to obtain CLX-NP.

#### 2.2.8 Preparation of DiR-labeled nanoparticles (DiR@LA-CLX-NP)

For *in vivo* imaging, near-infrared fluorescent dye DiR was incorporated into LA-CLX-NP. DiR (0.05 mg) was co-dissolved with LA-NP (5 mg) and celecoxib (0.25 mg) in chloroform-methanol (3:1, v/v, 5 mL), and the nanoparticles were prepared following the same thin-film hydration method as described in section 2.2.3.

#### 2.2.9 *In vivo* imaging and biodistribution

To evaluate the targeting capability of LA-CLX-NP to the injured intestine, intestinal I/R model mice were intravenously injected with DiR@LA-CLX-NP (equivalent to 185.2 mg/kg LA-NP). At 3, 6, 12, 24, and 48 h post-injection, mice were anesthetized and imaged using an *in vivo* imaging system (IVIS Spectrum, PerkinElmer) with excitation at 748 nm and emission at 780 nm. At 24 h post-injection, mice were euthanized, and major organs (heart, liver, spleen, lungs, kidneys) and intestinal tissues (injured and normal segments) were excised for *ex vivo* imaging. Fluorescence intensities were quantified using Living Image software.

#### 2.2.10 *In vitro* ROS scavenging assay with additional control groups

To dissect the contributions of ROS-responsive release and co-delivery synergy, RAW 264.7 cells were seeded in 12-well plates (2 × 10^5^ cells/well) and treated with LPS (100 ng/mL) for 12 h, followed by incubation with different formulations for 24 h. Intracellular ROS levels were detected using the DCFH-DA probe. Fluorescence images were captured under a fluorescence microscope, and quantitative analysis was performed by flow cytometry. Mean fluorescence intensity (MFI) and the percentage of ROS-positive cells were recorded.

#### 2.2.11 PGE2 ELISA

PGE2 levels in intestinal tissue homogenates were measured using a commercial ELISA kit according to the manufacturer's instructions. Briefly, intestinal tissues were homogenized in PBS containing protease inhibitors, and supernatants were collected after centrifugation. PGE2 concentrations were determined using a standard curve and normalized to total protein content (pg/mg protein) measured by BCA assay.

#### 2.2.12 Quantitative analysis of immunohistochemistry

Immunohistochemical staining for COX-2 was quantified using ImageJ software. For each section, five randomly selected non-overlapping high-power fields (200×) were analyzed. The positive area percentage was calculated as (positive area/total field area) × 100%. The relative COX-2 expression was normalized to the Sham group (set as 1.0).

#### 2.2.13 Chiu’s histological scoring

H&E-stained intestinal sections were evaluated blindly by two independent pathologists using Chiu's scoring system: 0, normal mucosa; 1, subepithelial space widening at the villous tip; 2, moderate separation of the epithelium from the lamina propria; 3, massive epithelial lifting along the villous sides; 4, denuded villi with dilated capillaries; 5, disintegration of the lamina propria, hemorrhage, and ulceration. The final score for each animal was the average of five randomly selected high-power fields.

#### 2.2.14 Acute toxicity and maximum tolerated dose (MTD) determination

To evaluate the safety window of LA-CLX-NP, an acute toxicity study was performed. Mice were randomly divided into seven dose groups (*n* = 10 per group): 185.2, 370.4, 740.8, 1,111.2, 1,481.6, 1,666.8, and 1,852.0 mg/kg (equivalent to 1-, 2-, 4-, 6-, 8-, 9-, and 10-fold of the therapeutic dose). A control group received an equal volume of saline. All formulations were administered via single tail vein injection, and mice were observed for 14 days. Body weight was recorded on days 0, 1, 3, 5, 7, 10, and 14. Mortality and signs of toxicity (e.g., reduced activity, piloerection, respiratory distress) were monitored daily. The MTD was defined as the highest dose causing no mortality and no severe toxicity (body weight loss ≤10% of control).

### 2.3 Cell experiments

#### 2.3.1 Cell culture

RAW264.7 cells were cultured in DMEM high-glucose medium supplemented with 10% FBS, 100 U/mL penicillin, and 100 µg/mL streptomycin at 37 °C in a 5% CO_2_ humidified incubator. Cells were subcultured every 2–3 days, and cells in the logarithmic growth phase were used for experiments.

#### 2.3.2 Cell viability assay (CCK-8)

RAW264.7 cells were seeded into 96-well plates at a density of 1 × 10^4^ cells/well and cultured overnight. The cells were then treated with various concentrations of free celecoxib, free α-lipoic acid, LA-NP, or LA-CLX-NP for 24 h. CCK-8 reagent (10 µL) was added to each well and incubated for 2 h. Absorbance was measured at 450 nm using a microplate reader.

#### 2.3.3 Cellular uptake assay

FITC was loaded onto LA-NP to obtain fluorescently labeled nanoparticles (FITC-LA-NP). RAW264.7 cells were seeded into confocal dishes and cultured overnight. The cells were then incubated with FITC-LA-NP (final concentration 1 µg/mL) at 37 °C for 2, 4, 6, or 8 h. After washing three times with PBS, the nuclei were stained with DAPI, and the cells were observed and photographed under a laser scanning confocal microscope.

#### 2.3.4 Evaluation of anti-inflammatory activity

RAW264.7 cells were seeded into 12-well plates at a density of 2 × 10^5^ cells/well. Except for the control group, cells were stimulated with LPS (100 ng/mL) for 24 h. The culture supernatants were collected for measurement of TNF-α, IL-6, and IL-10 by ELISA kits, as well as GSH, GSSG and SOD activities according to the manufacturers’ instructions. Intracellular ROS levels were determined using the DCFH-DA probe.

#### 2.3.5 Quantitative real-time PCR (qRT-PCR)

After the above treatments, total RNA was extracted using Trizol, and RNA concentration and purity were determined. One microgram of RNA was reverse-transcribed into cDNA. The qPCR reaction mixture contained SYBR Green Master Mix (10 µL), forward and reverse primers (0.4 µL each), cDNA template (2 µL), and ddH₂O to a final volume of 20 µL. The amplification program was: 95 °C for 2 min, followed by 40 cycles of 95 °C for 15 s, 60 °C for 30 s, and 72 °C for 30 s. GAPDH was used as an internal control, Primer sequences are listed in Supplementary Table S2.

### 2.4 Animal experiments

#### 2.4.1 Animals and grouping

SPF-grade male C57BL/6 mice (6–8 weeks old, 20–25 g) were acclimated for one week and then randomly divided into 5 groups (*n* = 5 per group):

Sham group: sham operation, laparotomy without vessel clamping. I/R group: intestinal I/R model without any treatment. CLX group: I/R model + tail vein injection of free celecoxib (10 mg/kg). LA-NP group: I/R model + tail vein injection of LA-NP (185.2 mg/kg). LA-CLX-NP group: I/R model + tail vein injection of LA-CLX-NP (185.2 mg/kg, equivalent to 10 mg/kg celecoxib).

The sample size of *n* = 5 per group was determined based on preliminary power analysis, which indicated that this number would provide sufficient statistical power (>80%) to detect meaningful differences in the primary endpoints (e.g., mechanical withdrawal threshold and inflammatory cytokine levels), while minimizing animal use in accordance with the 3R principles. All procedures were approved by the Animal Ethics Committee.

#### 2.4.2 Mouse intestinal ischemia-reperfusion model

Mice were fasted for 12 h with free access to water before surgery. Anesthesia was induced by intraperitoneal injection of pentobarbital sodium (40 mg/kg). A midline laparotomy was performed, and the superior mesenteric artery (SMA) was isolated. Ischemia was induced by clamping the SMA with a microvascular clamp. Successful ischemia was confirmed by paleness of the intestine and loss of peristalsis. After 45 min of ischemia, the clamp was removed to allow reperfusion, which was confirmed by restoration of normal color and peristalsis. The abdominal incision was then closed. In the sham group, the SMA was exposed but not clamped. Immediately after reperfusion, drugs or nanoparticles were administered via a single tail vein injection at a volume of 10 µL/g body weight.

#### 2.4.3 Pain-related behavioral assessments

All behavioral tests were performed at 4, 8, 12, 24, and 48 h after reperfusion by an investigator blinded to the group allocation.

Mechanical withdrawal threshold (von Frey test): Mice were placed in individual plexiglass chambers on a metal mesh grid and allowed to acclimate for 30 min. Von Frey filaments were applied perpendicularly to the abdominal skin starting from the lowest force. The minimal force that elicited a positive response (lifting, licking, or jumping of the abdomen) was recorded as the mechanical withdrawal threshold. Each mouse was tested three times, and the average value was taken.

Abdominal licking bouts: Mice were placed in a transparent plexiglass chamber. After 10 min of acclimation, the number of abdominal licking episodes was counted over a 10-min period. Abdominal licking was defined as the mouse lifting its hind leg, turning its head toward the abdomen, and licking the abdominal area.

Scratching bouts: During the same observation period, the number of scratching episodes directed to the abdomen or flank was recorded. One continuous scratching movement (e.g., three consecutive scratches) was counted as one bout.

Diarrhea score: Fecal status was observed at 4, 8, 12, 24, and 48 h after reperfusion and scored as follows: 0 = normal formed stool or slightly soft stool; 1 = pasty or watery stool. Diarrhea incidence (%) = (number of mice with a score of 1/total number of mice in the group) × 100%.

#### 2.4.4 Sample collection and processing

Mice were euthanized at 48 h post-reperfusion (or at the indicated time points) by intraperitoneal injection of an overdose of pentobarbital sodium (150 mg/kg), followed by cervical dislocation to confirm death. Blood was collected from the orbital sinus, allowed to clot, and then centrifuged at 3,000 rpm for 10 min to obtain serum, which was stored at −80 °C. The small intestinal tissue (the central segment of the ischemic region, approximately 3–4 cm in length) was rapidly excised, opened longitudinally along the anti-mesenteric border, and gently rinsed with ice-cold PBS. The tissue was divided into three portions: one portion was fixed in 4% paraformaldehyde for histopathological and immunohistochemical analyses; the remaining two portions were immediately homogenized and centrifuged, and the supernatants were used for ELISA and oxidative stress marker measurements.

#### 2.4.5 Immunohistochemistry (IHC)

Paraffin-embedded sections were deparaffinized and rehydrated. Antigen retrieval was performed by microwave heating in citrate buffer (pH 6.0). Endogenous peroxidase was blocked with 3% H_2_O_2_ and non-specific binding was blocked with 5% BSA. Sections were incubated with anti-COX-2 primary antibody (1:200) at 4 °C overnight, followed by HRP-conjugated secondary antibody. Staining was developed with DAB, and nuclei were counterstained with hematoxylin. Images were captured under a light microscope, and the percentage of positive cells and staining intensity were analyzed using ImageJ software.

#### 2.4.6 Immunofluorescence (IF)

Fixed intestinal tissues were embedded in paraffin and cut into 5-µm thick sections. Sections were deparaffinized, rehydrated, and subjected to antigen retrieval in citrate buffer (pH 6.0) using microwave heating. After cooling, sections were blocked with 5% BSA for 30 min at room temperature, then incubated with anti-ZO-1 primary antibody (1:200) at 4 °C overnight. After washing with PBS, sections were incubated with Alexa Fluor 488-conjugated secondary antibody (1:500) for 1 h at room temperature in the dark. After washing, the sections were mounted with DAPI-containing mounting medium. Images were captured using a laser scanning confocal microscope (ZO-1: green fluorescence; nuclei: blue).

#### 2.4.7 Measurement of inflammatory cytokines and oxidative stress markers

Serum and intestinal tissue homogenate supernatants were used to measure TNF-α, IL-6, and IL-1β levels by ELISA kits. MDA content, SOD activity, and GSH content in intestinal homogenates were determined according to the respective kit instructions.

#### 2.4.8 Serum biochemical analysis

Blood samples were collected at 48 h post-reperfusion, and serum was separated by centrifugation. Serum levels of alanine aminotransferase (ALT), aspartate aminotransferase (AST), blood urea nitrogen (BUN), and creatinine (CR) were measured using an automatic biochemical analyzer (Hitachi 7180) according to the manufacturer's protocols.

#### 2.4.9 *In vivo* imaging and biodistribution

To verify the intestinal targeting of LA-CLX-NP, mice were intravenously injected with DiR@LA-CLX-NP (185.2 mg/kg) immediately after reperfusion. *In vivo* fluorescence imaging was performed at 3, 6, 12, 24, and 48 h post-injection using an IVIS imaging system. At 24 h post-injection, mice were euthanized, and major organs and intestinal segments were collected for *ex vivo* imaging to assess nanoparticle distribution.

### 2.5 Statistical analysis

All data are expressed as mean ± standard deviation (SD). Statistical analyses were performed using GraphPad Prism 9.0. Comparisons among multiple groups were carried out by one-way ANOVA followed by Tukey's *post-hoc* test. Two-way ANOVA with Bonferroni's multiple comparisons test was used for data with multiple time points. Diarrhea incidence was analyzed using the chi-square test. A value of *p < 0.05 was considered statistically significant.

## 3 Results

### 3.1 Synthesis and characterization of LA-CLX-NP

The LA-NP copolymer was successfully synthesized via an amidation reaction. ¹H NMR spectra (Supplementary Figures S1, S2) showed that the carboxylic proton of free LA appeared as a broad singlet at approximately δ 12.0, whereas this signal completely disappeared in the LA-NP spectrum, while the characteristic peaks of the DSPE-PEG backbone were clearly visible. These results confirmed that the carboxyl group of LA was fully consumed during the amidation reaction, indicating successful conjugation of LA to the polymer backbone.

Celecoxib-loaded nanoparticles (LA-CLX-NP) were prepared by the thin-film hydration method. UV-Vis absorption spectra (Figure 1A) showed that free CLX exhibited a characteristic absorption peak at 254 nm, which was absent in DSPE-PEG-NH₂, while LA-CLX-NP displayed the same absorption peak, indicating successful loading of CLX. ¹H NMR spectra (Supplementary Figures S3, S4) further verified the successful construction of LA-CLX-NP, as proton signals corresponding to the tolyl and benzenesulfonamide groups of celecoxib appeared at δ 7–8 ppm in the LA-CLX-NP spectrum. Integration of the LA ring proton signals (δ 3.1–3.2 ppm) relative to the DSPE terminal methyl signal (*δ* 0.88 ppm) yielded an LA grafting efficiency of approximately 89.9%, indicating that the majority of DSPE-PEG-NH₂ molecules were successfully conjugated with LA. DLS analysis showed that LA-CLX-NP had a mean hydrodynamic diameter of 146.0 ± 1.57 nm with a polydispersity index (PDI) of 0.15 ± 0.01 (Figure 1B), indicating a uniform size distribution. TEM images (Figure 1C) revealed spherical nanoparticles with homogeneous morphology, consistent with the DLS results. The drug loading of CLX was determined to be 2.8%, and the encapsulation efficiency was 54.0%. When incubated in 10% FBS at 37 °C for 7 days, the particle size remained almost constant (Figure 1D), demonstrating good colloidal stability of LA-CLX-NP under physiological conditions.

**Figure 1:**
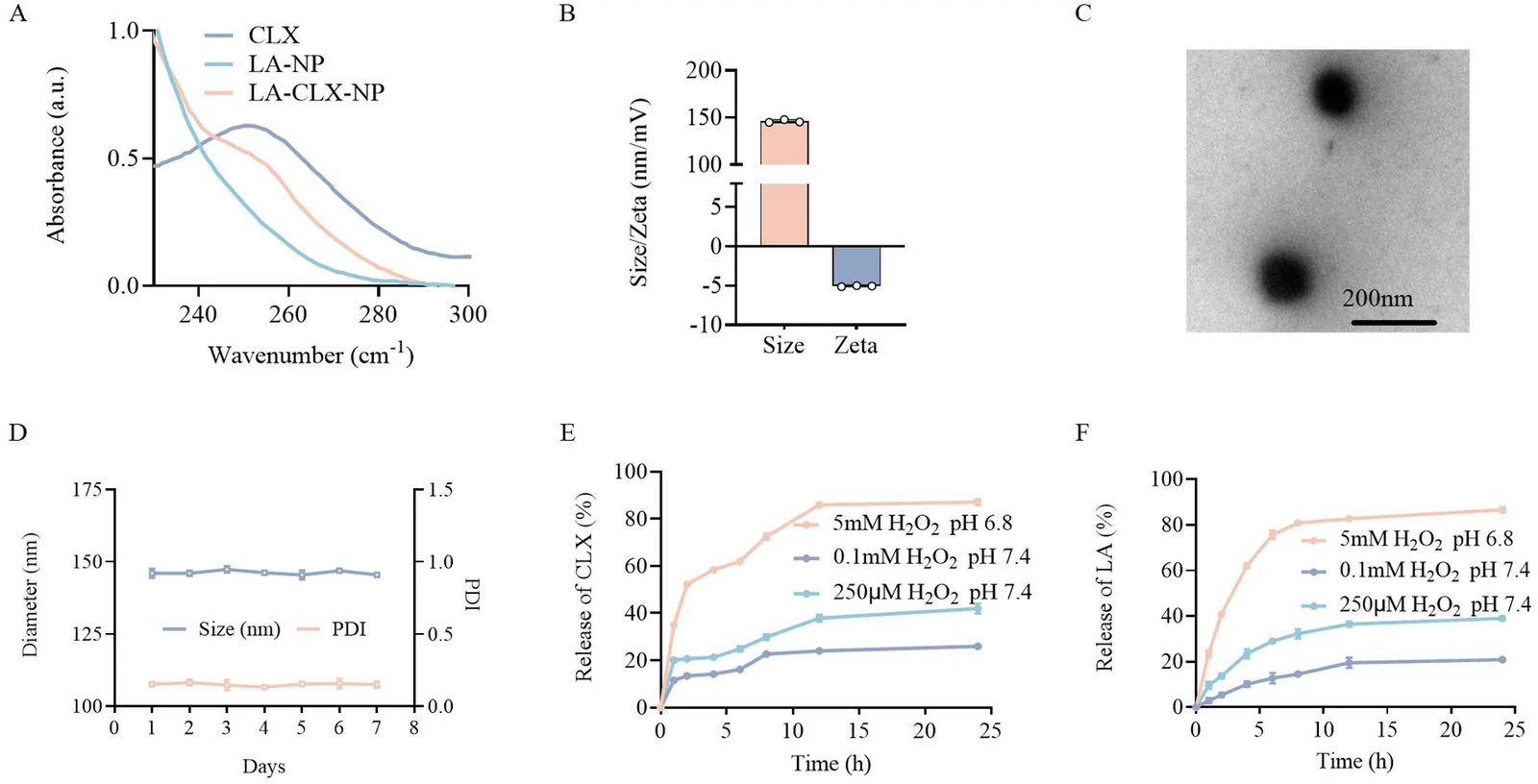
Synthesis and characterization of LA-CLX-NP. **(A)** UV-Vis of free CLX, DSPE-PEG-NH_2_, and LA-CLX-NP. **(B)** Hydrodynamic diameter distribution (DLS) and zeta potential of LA-CLX-NP. **(C)** Representative TEM image of LA-CLX-NP (scale bar = 200 nm). **(D)** Colloidal stability of LA-CLX-NP in 10% FBS at 37 °C over 7 days (data shown as mean ± SD, *n* = 3). **(E,F)** Cumulative release profiles of CLX **(E)** and LA **(F)** from LA-CLX-NP under different conditions: PBS (pH 7.4) without H_2_O_2_, PBS (pH 7.4) with 0.1 mM H_2_O_2_, PBS (pH 7.4) with 250 µM H_2_O_2_, and PBS (pH 6.8) with 5 mM H_2_O_2_. Data are presented as mean ± SD (*n* = 3). Data are presented as mean ± SD (*n* = 3).

The ROS-responsive drug release behavior of LA-CLX-NP was investigated using a dialysis method. Under mild oxidative conditions (PBS, pH 7.4, 0.1 mM H_2_O_2_), the cumulative release of CLX remained relatively low, reaching 25.95% at 24 h (Figure 1E). When the H₂O₂ concentration was increased to 0.25 mM (PBS, pH 7.4), the release increased to 41.94% at 24 h. Notably, when the release medium was changed to an acidic pH (6.8) combined with a higher H_2_O_2_ concentration (5 mM), the release was markedly enhanced, reaching 87.06% at 24 h. The release of LA was assessed in parallel under the same conditions (Figure 1F), showing 24-h cumulative release rates of 20.91% (pH 7.4, 0.1 mM H_2_O_2_), 38.97% (pH 7.4, 0.25 mM H_2_O_2_), and 86.64% (pH 6.8, 5 mM H_2_O_2_), respectively. These pH- and ROS-dependent release profiles confirm the dual-responsive nature of the nanoplatform, with the enhanced release being specifically mediated by the cleavage of disulfide bonds in LA under oxidative conditions.

### 3.2 *In vitro* cytotoxicity, cellular uptake, and anti-inflammatory activity of LA-CLX-NP

The cytotoxicity of CLX, LA, and LA-CLX-NP against RAW 264.7 cells was evaluated by the CCK-8 assay. As shown in Figure 2A, both CLX and LA inhibited cell viability in a concentration-dependent manner. LA maintained cell viability above 80% across the tested concentration range (0.5–640 µM), indicating low toxicity, whereas CLX caused a marked decrease in viability at high concentrations (≥200 µM). Further assessment of LA-CLX-NP cytotoxicity (Figure 2B) showed that within the equivalent CLX concentration range of 0.5–32 µM, cell viability remained above 85% and was not significantly different from the control group, indicating good biocompatibility of the nanocarrier.

**Figure 2:**
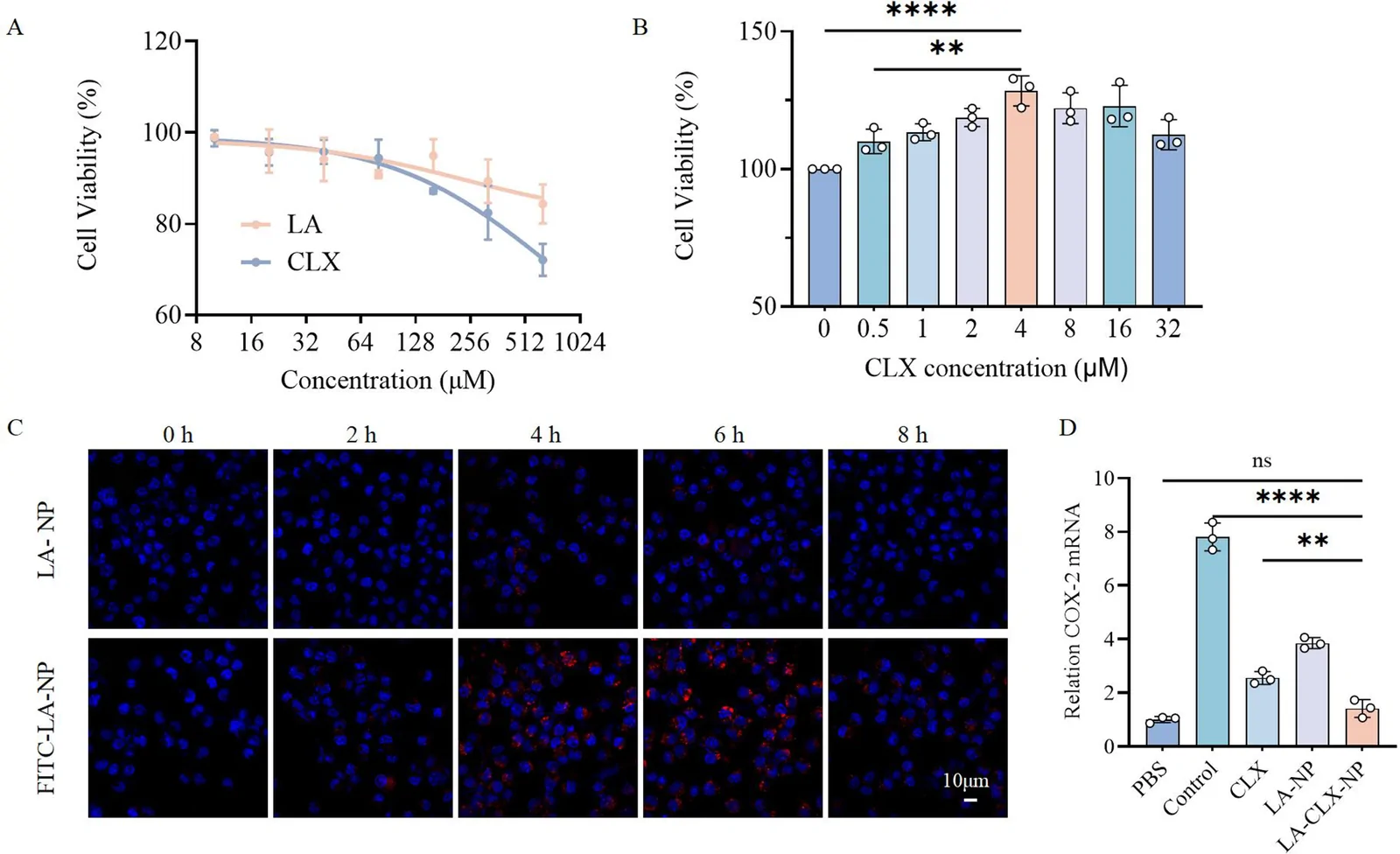
*In vitro* cytotoxicity, cellular uptake, and anti-inflammatory activity of LA-CLX-NP. **(A)** Cell viability of RAW 264.7 cells after 24 h treatment with various concentrations of CLX or LA. **(B)** Cell viability of RAW 264.7 cells after 24 h treatment with increasing concentrations of LA-CLX-NP (expressed as equivalent CLX concentration). **(C)** Time-dependent uptake of FITC-labeled nanoparticles (FITC-LA-NP) in RAW 264.7 cells. Scale bar = 10 µm. **(D)** Relative COX-2 mRNA expression in LPS-stimulated RAW 264.7 cells after treatment with the indicated formulations (CLX, LA-NP, and LA-CLX-NP). Data are shown as mean ± SD (*n* = 3). *p < 0.05, **p < 0.01, ***p < 0.001 vs. control or as indicated.

The cellular uptake of nanoparticles was examined using FITC-labeled LA-NP (FITC-LA-NP). Fluorescence images (Figure 2C) showed that intracellular green fluorescence intensity gradually increased with prolonged co-incubation time (0–6 h), with obvious fluorescence aggregation observed at 6 h, indicating efficient internalization of the nanoparticles by macrophages in a time-dependent manner.

The expression level of COX-2 mRNA in LPS-stimulated RAW 264.7 cells was detected by qRT-PCR. As shown in Figure 2D, LPS stimulation significantly upregulated COX-2 mRNA expression compared with the control group. Treatment with free CLX or LA-NP alone partially reduced COX-2 expression, whereas the LA-CLX-NP group exhibited the most pronounced inhibition, with COX-2 mRNA levels approaching those of the control group and significantly better than those in the free CLX and LA-NP groups. These results suggest that LA-CLX-NP, through the synergistic delivery of CLX and LA, can more effectively suppress the transcription of the key inflammatory enzyme COX-2, providing an *in vitro* basis for its subsequent *in vivo* anti-inflammatory and analgesic effects.

### 3.3 Inhibitory effect of LA-CLX-NP on ROS levels in LPS-stimulated RAW 264.7 cells

Intracellular ROS levels were measured using the DCFH-DA fluorescent probe. As shown in Figures 3A,B, LPS stimulation markedly increased green fluorescence intensity (model group), indicating successful induction of oxidative stress. After treatment with free CLX or LA-NP, the fluorescence intensity decreased compared with the model group, while the LA-CLX-NP group exhibited the weakest fluorescence, closest to the control group. Quantitative analysis of fluorescence intensity (Figure 3B) showed that the mean fluorescence intensity in the model group was 154.42 ± 3.92 a.u., significantly higher than that in the control group. Both free CLX and LA-NP groups showed significantly lower fluorescence than the model group, and the LA-CLX-NP group further reduced it to 76.94 ± 7.05 a.u., which was significantly better than both single-agent groups.

**Figure 3:**
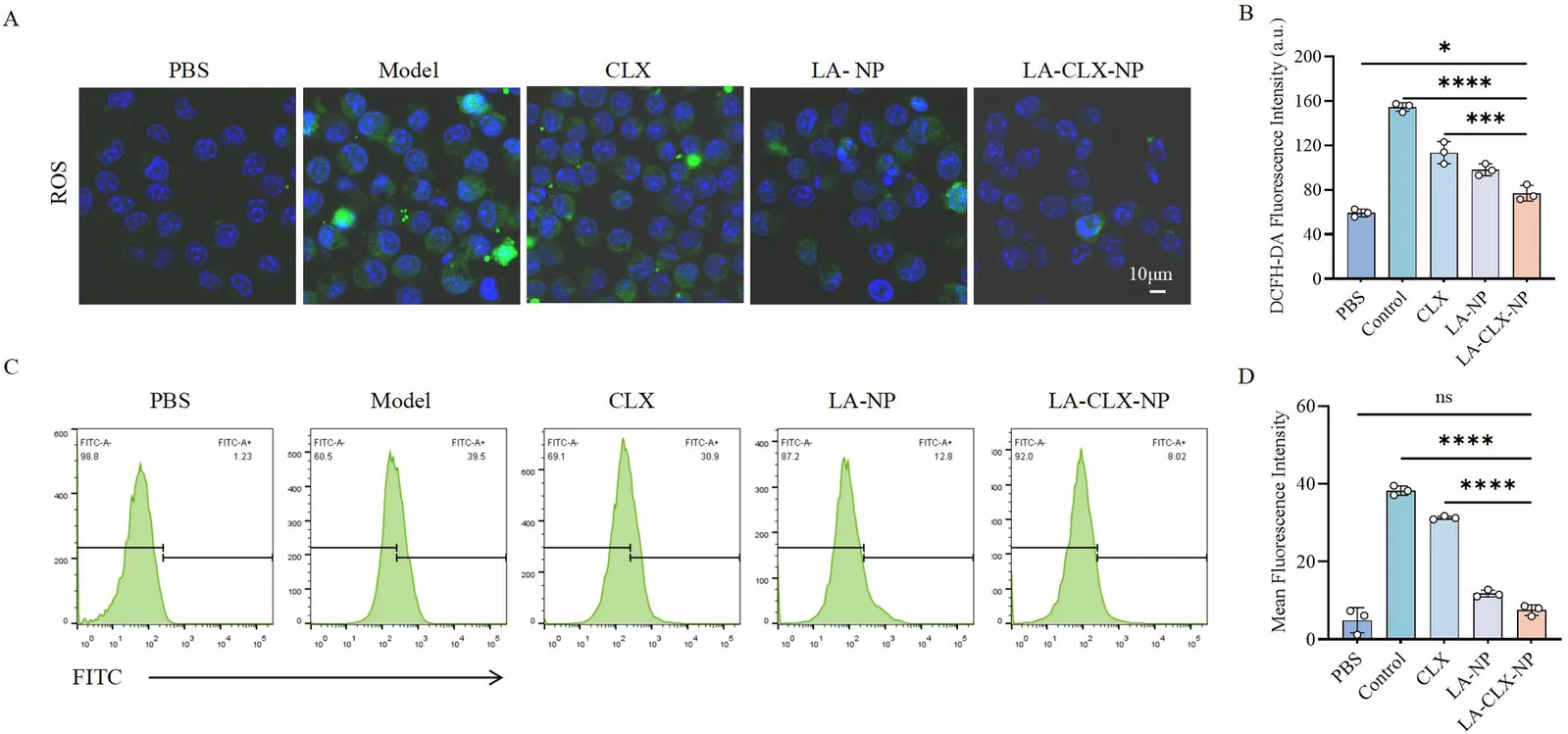
Inhibitory effect of LA-CLX-NP on ROS levels in LPS-stimulated RAW 264.7 cell. **(A)** Representative fluorescence images of intracellular ROS levels detected by the DCFH-DA probe. Scale bar = 10 µm. **(B)** Quantitative analysis of cellular ROS fluorescence intensity. **(C)** Flow cytometry histograms of intracellular ROS fluorescence intensity. **(D)** Quantification of mean fluorescence intensity from flow cytometry. Data are presented as mean ± SD (*n* = 3). *p < 0.05, **p < 0.01, ***p < 0.001 vs. control or as indicated.

Flow cytometry analysis (Figure 3C) revealed a marked rightward shift of the fluorescence peak after LPS stimulation, whereas treatment with LA-CLX-NP caused a clear leftward shift, approaching the control level. Quantitative results (Figure 3D) showed that the percentage of positive cells was 38.27% in the model group, decreased to 11.80% in the LA-NP group, and further decreased to 7.53% in the LA-CLX-NP group, which was not significantly different from the control group (4.92%). Notably, the LA-CLX-NP group exhibited the weakest fluorescence, closest to the control group, and outperformed the free CLX + LA combination and the non-responsive CLX-NP control groups (Supplementary Figures S5, S6), confirming the essential roles of ROS-responsive release and co-delivery synergy. Collectively, these results consistently demonstrate that LA-CLX-NP efficiently scavenges LPS-induced intracellular ROS in macrophages through the synergistic delivery of CLX and LA, providing a strong *in vitro* basis for its *in vivo* antioxidant protective effects.

### 3.4 Regulation of oxidative stress and inflammatory cytokines by LA-CLX-NP in LPS-stimulated RAW 264.7 cells

The levels of total glutathione (T-GSH), oxidized glutathione (GSSG), superoxide dismutase (SOD) activity in RAW 264.7 cells, as well as IL-10, IL-6, and TNF-α in the culture supernatant, were further measured after LPS stimulation. As shown in Figure 4A, compared with the PBS group, LPS stimulation dramatically decreased intracellular T-GSH content from 7.43 ± 0.22 nmol/10^6^ cells to 0.82 ± 0.08 nmol/10^6^ cells. Free CLX treatment partially restored T-GSH, and the LA-CLX-NP group further elevated it to 5.66 ± 0.27 nmol/10^6^ cells, significantly higher than the model group. GSSG levels (Figure 4B) showed the opposite trend: the model group had significantly higher GSSG than the control group, free CLX lowered it, and the LA-CLX-NP group reduced GSSG to the lowest level (0.58 ± 0.08 nmol/10^6^ cells). SOD activity (Figure 4C) was significantly decreased in the model group compared with the control group; both free CLX and LA-CLX-NP restored SOD activity to different extents, with the LA-CLX-NP group showing the highest activity (26.15 ± 2.48 U/mg prot), significantly higher than the model group.

**Figure 4:**
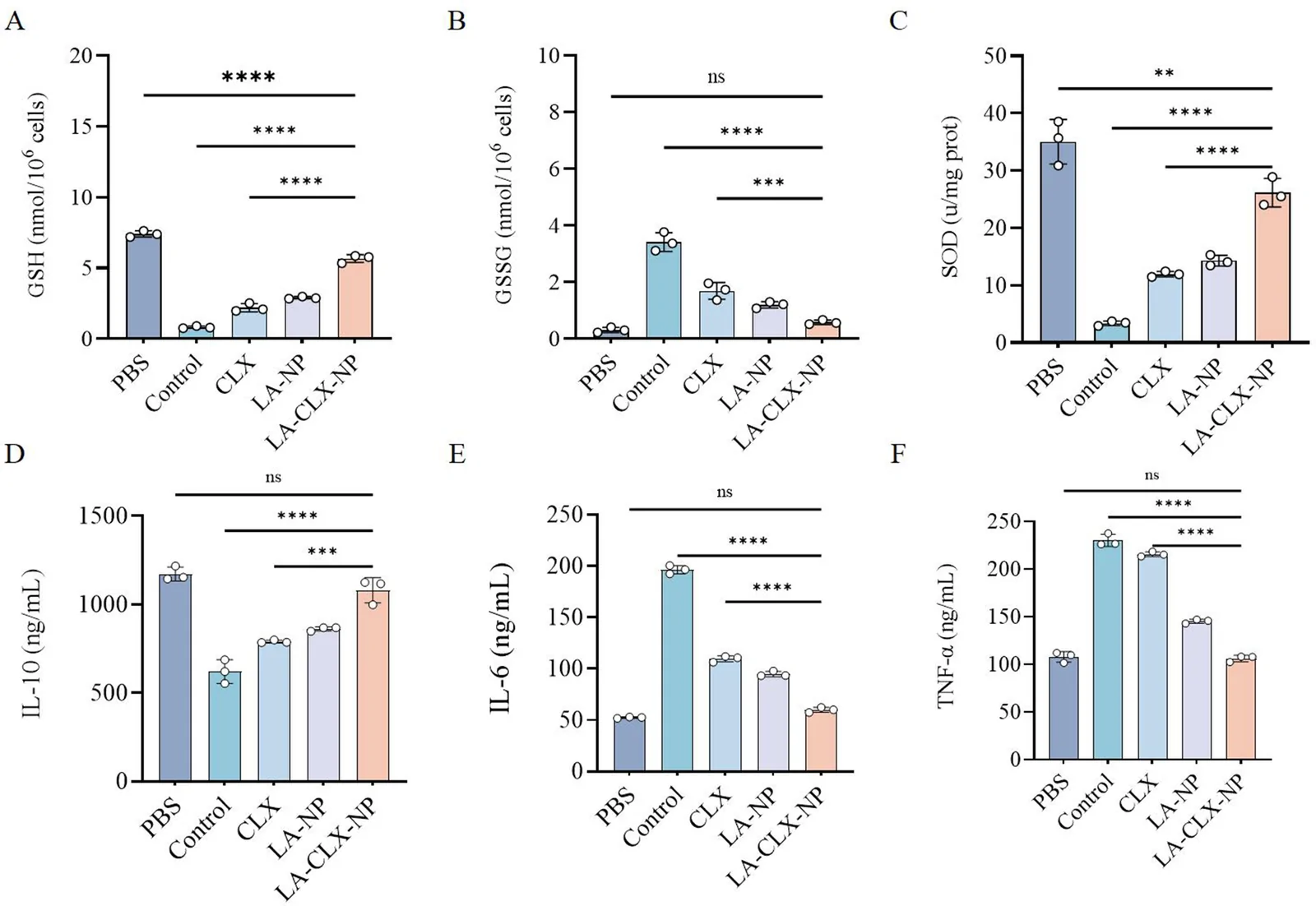
Effects of LA-CLX-NP on oxidative stress and inflammatory cytokine levels in LPS-stimulated RAW 264.7 macrophages. (A) Total glutathione (GSH) levels, (B) oxidized glutathione (GSSG) levels, (C) superoxide dismutase (SOD) activity, (D) IL-10 levels, (E) IL-6 levels, and (F) TNF-α levels in LPS-stimulated RAW 264.7 macrophages after treatment with the indicated formulations. Data are shown as mean ± SD (*n* = 3).*p < 0.05, **p < 0.01, ***p < 0.001 vs. control or as indicated.

Inflammatory cytokine measurements (Figures 4D–F) showed that LPS stimulation significantly decreased the anti-inflammatory cytokine IL-10 and markedly increased the pro-inflammatory cytokines IL-6 and TNF-α. Treatment with free CLX or LA-NP partially reversed these changes; the LA-CLX-NP group exhibited significantly better effects: IL-10 levels were significantly higher than those in the model group, and IL-6 and TNF-α levels were significantly lower than those in the model group and the free CLX group (*P* < 0.001). Notably, IL-6 and TNF-α levels in the LA-CLX-NP group were not statistically different from those in the control group, indicating efficient suppression of the inflammatory response. In summary, LA-CLX-NP, through the synergistic delivery of CLX and LA, significantly restored LPS-induced redox imbalance (increased T-GSH, decreased GSSG, restored SOD activity) and effectively modulated the inflammatory cytokine network (increased IL-10, decreased IL-6/TNF-α), demonstrating superior anti-inflammatory and antioxidant synergistic effects compared with single agents.

### 3.5 Effects of LA-CLX-NP on pain behaviors and gastrointestinal function in a mouse intestinal I/R model

To verify the targeting capability of LA-CLX-NP to the injured intestine, we performed *in vivo* imaging in intestinal I/R model mice using near-infrared fluorescent dye DiR-labeled LA-CLX-NP (DiR@LA-CLX-NP). As shown in Figure 5A, following intravenous administration, DiR@LA-CLX-NP exhibited a distinct fluorescent signal accumulation in the abdominal region as early as 3 h post-reperfusion, with signal intensity reaching its peak at 24 h and persisting up to 48 h. *Ex vivo* imaging of isolated organs (Figures 5B,C) revealed that the fluorescence intensity in the injured intestinal segment was markedly higher than that in the normal intestinal segment, as well as in the heart, liver, spleen, lungs, and kidneys. These findings provide direct evidence that LA-CLX-NP preferentially accumulates in the I/R-injured intestinal tissue, thereby supporting the on-demand delivery capability of this nanoplatform. Based on the above *in vitro* synergistic anti-inflammatory and antioxidant effects, we further evaluated the *in vivo* analgesic efficacy of LA-CLX-NP and its impact on gastrointestinal function in a mouse intestinal I/R model (Figure 5D). Diarrhea is a direct manifestation of intestinal barrier dysfunction after I/R injury (27). As shown in Figure 5E, no diarrhea was observed in the sham group. In the I/R group, the diarrhea incidence reached 100% at 4 h after reperfusion, remained 100% at 12 h, decreased to 80% at 24 h, and to 60% at 48 h. The free CLX group showed a reduced but still high incidence, and the LA-NP group exhibited a similar trend. In contrast, the LA-CLX-NP group had a significantly reduced diarrhea incidence of 40% at 4 h, 20% at 12 h, and complete disappearance (0%) after 24 h. These results indicate that LA-CLX-NP rapidly repairs the intestinal barrier through synergistic anti-inflammatory and antioxidant actions, effectively controls diarrhea, and promotes early recovery of gastrointestinal function.

**Figure 5:**
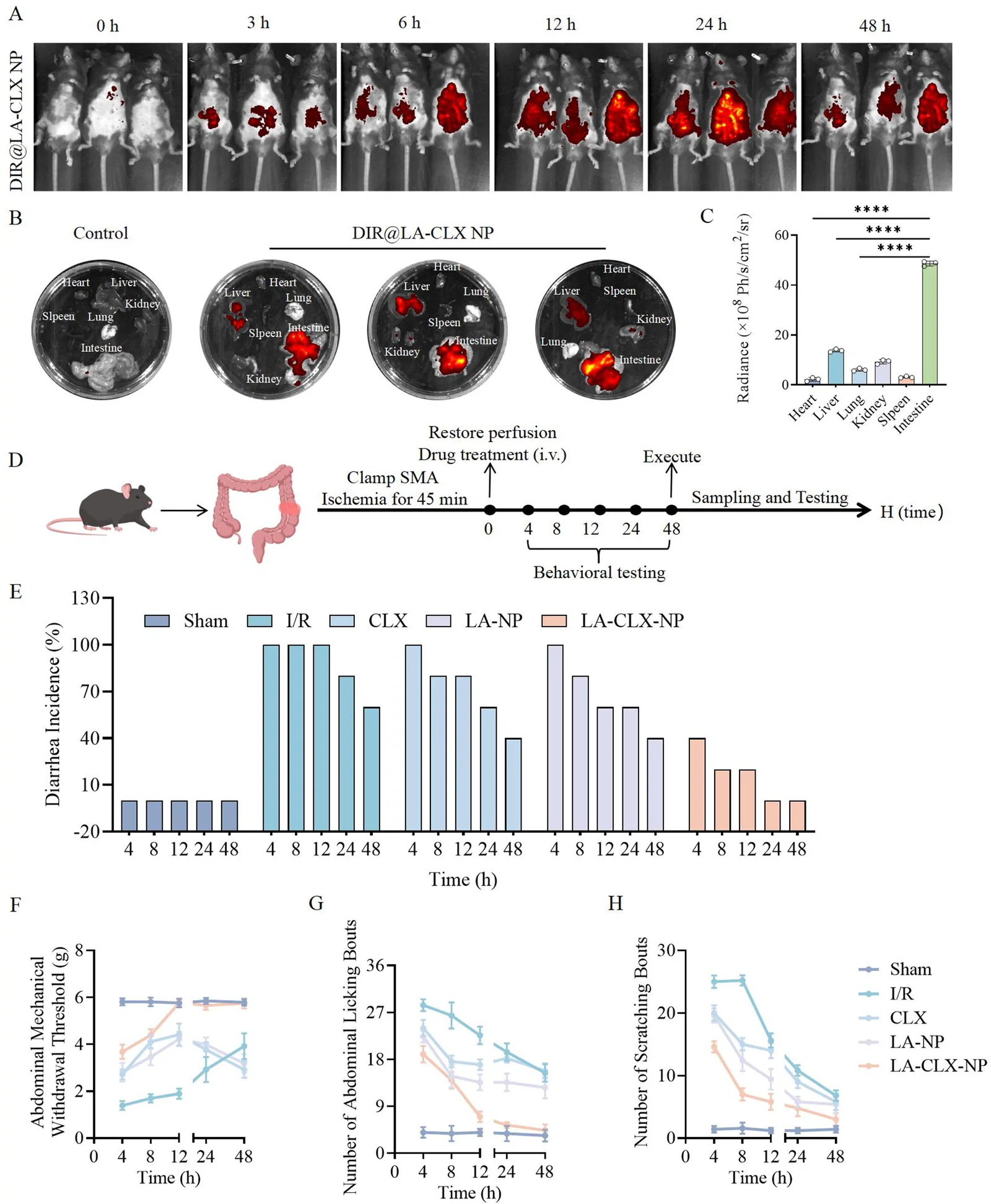
Effects of LA-CLX-NP on pain-related behaviors and gastrointestinal function in a mouse model of intestinal ischemia-reperfusion (I/R) injury. (A) *in vivo* fluorescence imaging of mice at various time points (3, 6, 12, 24, and 48 h) after intravenous injection of DiR@LA-CLX-NP. (B) *ex vivo* fluorescence imaging of major organs (heart, liver, spleen, lungs, kidneys) and intestinal tissues harvested at 24 h post-injection. (C) Quantitative analysis of fluorescence intensity in isolated organs and intestinal tissues. (D) Schematic illustration of the mouse intestinal I/R model. (E) Diarrhea incidence at different time points after reperfusion (4, 8, 12, 24, 48 h). (F) Abdominal mechanical withdrawal threshold measured by von Frey filaments. (G) Number of abdominal licking bouts (spontaneous visceral pain). (H) Number of scratching bouts (neurogenic inflammation). Data are presented as mean ± SD (*n* = 5 per group). *p < 0.05, **p < 0.01, ***p < 0.001 vs. I/R group.

The abdominal mechanical withdrawal threshold was measured using von Frey filaments (Figure 5F). The sham group maintained a stable threshold. In the I/R group, the threshold dropped significantly to 1.39 ± 0.19 g at 4 h after reperfusion, remained low from 12 to 24 h, and partially recovered by 48 h. The free CLX and LA-NP groups showed slightly higher thresholds than the I/R group, but the effect waned after 24 h. In contrast, the LA-CLX-NP group had a significantly higher threshold than the I/R group at 4 h (3.68 ± 0.30 g), reached a peak at 12 h (5.77 ± 0.14 g) close to the sham level, and maintained this effect up to 48 h. These data demonstrate that LA-CLX-NP provides rapid and long-lasting relief from mechanical allodynia induced by intestinal I/R, with efficacy significantly superior to single agents.

Abdominal licking behavior reflects spontaneous visceral pain (Figure 5G). The I/R group showed the highest number of licking bouts at 4 h (28.4 ± 1.14 bouts/10 min), which remained high at 12 h. Treatment with free CLX or LA-NP reduced licking counts, but a slight rebound occurred after 24 h. The LA-CLX-NP group significantly decreased licking bouts to 19.0 ± 1.58 at 4 h and further to 7.0 ± 1.0 at 12 h, approaching sham levels at 24 h, with effects significantly better than those of the single agents.

Scratching behavior is a specific indicator of neurogenic inflammation (Figure 5H). The I/R group exhibited the highest number of scratching bouts at 8 h after reperfusion (25.2 ± 0.84 bouts/10 min). Free CLX or LA-NP alone partially reduced scratching, whereas the LA-CLX-NP group markedly decreased scratching (14.6 ± 0.89 at 4 h; 5.8 ± 1.30 at 12 h), approaching sham levels by 24 h. This indicates that LA-CLX-NP effectively blocks neurogenic inflammation by scavenging ROS and inhibiting the release of inflammatory cytokines. Collectively, LA-CLX-NP significantly reduced diarrhea incidence, increased mechanical withdrawal threshold, and decreased spontaneous abdominal licking and scratching behaviors in the intestinal I/R model, achieving a unification of potent analgesia and rapid gastrointestinal functional recovery.

### 3.6 Protective effect of LA-CLX-NP on intestinal barrier function in the mouse intestinal I/R model

To further elucidate the protective mechanism, we examined the expression and distribution of COX-2 and ZO-1 in intestinal tissue, as well as serum levels of pro-/anti-inflammatory cytokines. Immunohistochemical staining (Figure 6A) showed that COX-2 expression was weakly positive in the intestinal mucosa of the sham group. In the I/R group, COX-2-positive cells were markedly increased, with intense brown staining widely distributed in the villous epithelium and inflammatory cells of the lamina propria, consistent with the severe behavioral phenotype. After treatment with free CLX or LA-NP, the number of positive cells decreased but remained relatively high. The LA-CLX-NP group exhibited the fewest COX-2-positive cells, with staining intensity close to that of the sham group, indicating that this nanoparticle efficiently suppresses COX-2 overexpression, thereby reducing the generation of pain- and inflammation-related mediators at the source. Quantitative immunohistochemical analysis (Figure 6B) revealed that COX-2 expression in the I/R group was significantly upregulated to 2.39 ± 0.06 relative to the Sham group. Treatment with free CLX or LA-NP partially reduced COX-2 expression, whereas the LA-CLX-NP group exhibited a marked reduction to levels approaching those of the Sham group, further demonstrating that this nanoparticle effectively suppresses COX-2 overexpression, thereby attenuating the generation of pain- and inflammation-related mediators at the source.

**Figure 6:**
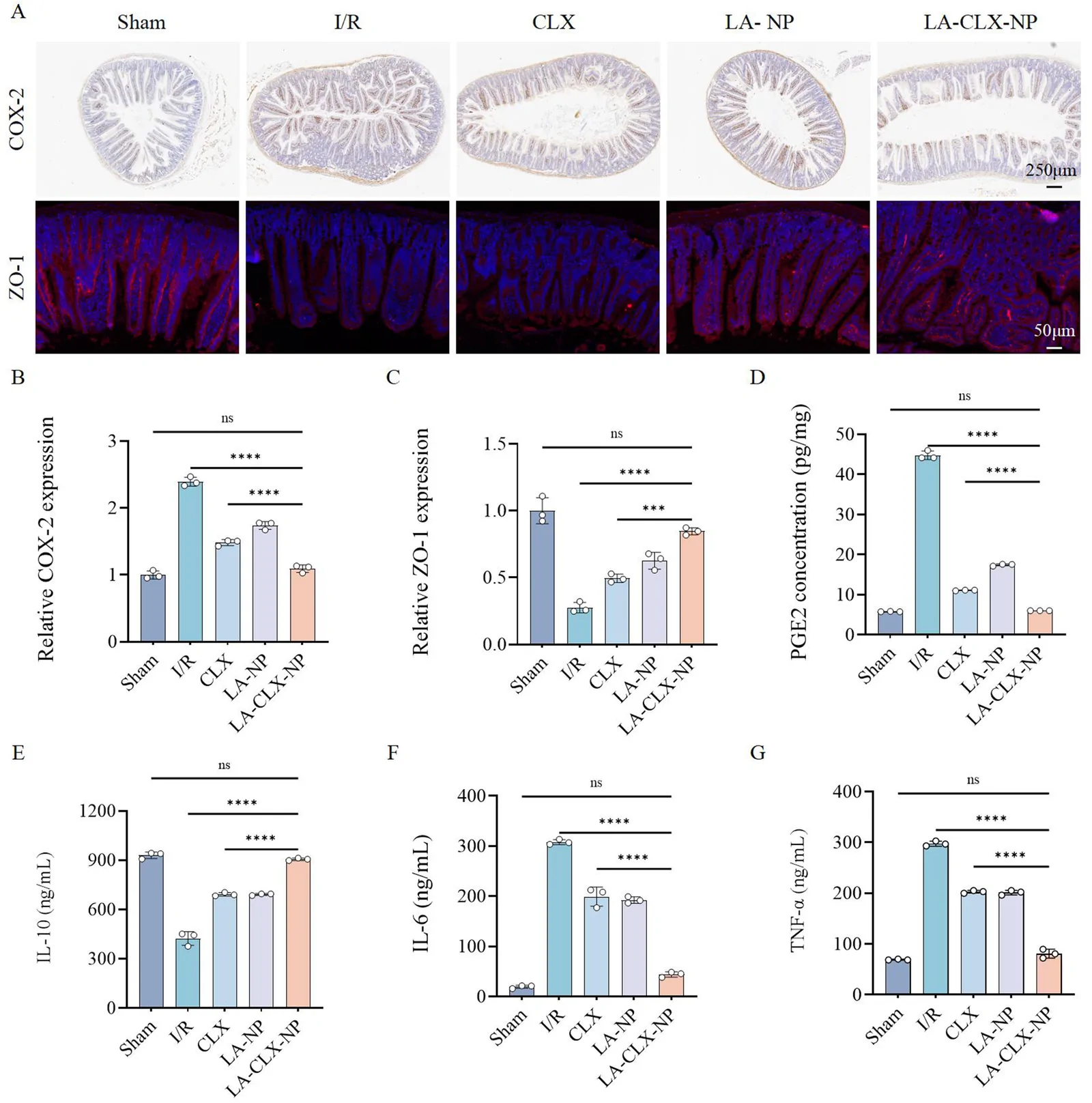
Protective effect of LA-CLX-NP on intestinal barrier function in a mouse model of intestinal ischemia-reperfusion (I/R) injury. **(A)** Representative images of COX-2 immunohistochemistry and ZO-1 immunofluorescence in intestinal tissues from the indicated treatment groups. **(B)** Quantitative analysis of relative COX-2 expression based on immunohistochemical staining. **(C)** Quantitative analysis of relative ZO-1 expression normalized to the Sham group, based on immunofluorescence intensity. **(D)** PGE2 levels in intestinal tissue homogenates measured by ELISA. **(E)** Serum TNF-α levels, **(F)** serum IL-6 levels, and **(G)** serum IL-10 levels. Data are shown as mean ± SD (*n* = 3).*p < 0.05, **p < 0.01, ***p < 0.001 vs. I/R group.

Immunofluorescence staining of ZO-1 (Figure 6A) showed that in the Sham group, ZO-1 was continuously and regularly distributed along the intestinal epithelial cell membrane in a characteristic “chicken-wire” pattern. In the I/R group, ZO-1 staining was markedly fragmented, sparse, and discontinuous, indicating severe disruption of tight junctions, consistent with the high incidence of diarrhea and impaired intestinal barrier function. Treatment with free CLX or LA-NP partially restored ZO-1 continuity, although interruptions remained evident. In contrast, the LA-CLX-NP group exhibited continuous and intact ZO-1 staining, morphologically similar to that of the Sham group. Quantitative analysis (Figure 6C) demonstrated that ZO-1 fluorescence intensity in the LA-CLX-NP group was significantly increased compared with the I/R group and was superior to that of either single-agent treatment group.

Furthermore, PGE2 levels in intestinal tissue homogenates were measured by ELISA (Figure 6D). PGE2 concentration was markedly elevated in the I/R group, while treatment with free CLX or LA-NP partially reduced PGE2 levels. Notably, the LA-CLX-NP group showed the most pronounced reduction, with PGE2 concentration decreased to 6.03 ± 0.07 pg/mg protein, which was significantly lower than that in the I/R group and the single-agent treatment groups.

Serum cytokine measurements (Figures 6E–6G) showed that the I/R group had significantly elevated TNF-α and IL-6 levels and significantly decreased IL-10 levels compared with the sham group. The LA-CLX-NP group reduced TNF-α and IL-6 to 80.70 ± 8.67 ng/mL and 44.12 ± 5.27 ng/mL, respectively, which were significantly lower than those in the I/R group and the single-agent groups; simultaneously, IL-10 levels were increased to 906.21 ± 7.51 ng/mL, significantly higher than in the model and single-agent groups, and were not significantly different from the sham group. These results indicate that LA-CLX-NP effectively suppresses systemic pro-inflammatory responses and restores the pro-/anti-inflammatory balance, providing a molecular basis for its pain-relieving and gastrointestinal-recovery-promoting effects.

Overall, LA-CLX-NP exerts synergistic anti-inflammatory, barrier-protective, and analgesic effects in intestinal ischemia-reperfusion injury by suppressing COX-2 expression and subsequent PGE2 production, preserving the ZO-1-mediated intestinal epithelial barrier, and modulating the systemic cytokine profile—downregulating serum TNF-α and IL-6 levels while upregulating IL-10. Its therapeutic efficacy is significantly superior to that of free CLX or LA-NP alone.

### 3.7 *In vivo* safety evaluation of LA-CLX-NP

To systematically evaluate the safety profile of LA-CLX-NP, we performed histopathological examination of intestinal tissues, serum biochemical analysis, and acute toxicity testing to determine the maximum tolerated dose (MTD).

Histopathological evaluation by H&E staining (Figure 7A) revealed that the Sham group exhibited intact mucosal architecture with well-arranged villi and no obvious pathological alterations. In contrast, the I/R group displayed severe mucosal injury, characterized by villus disruption, epithelial shedding, and extensive inflammatory cell infiltration in the lamina propria. The free CLX group showed moderate mucosal damage with partial villus recovery, although notable structural abnormalities persisted. The LA-NP group demonstrated improved mucosal integrity with only mild edema. Remarkably, the LA-CLX-NP group exhibited nearly intact mucosal architecture with regularly arranged villi, morphologically comparable to the Sham group.

**Figure 7:**
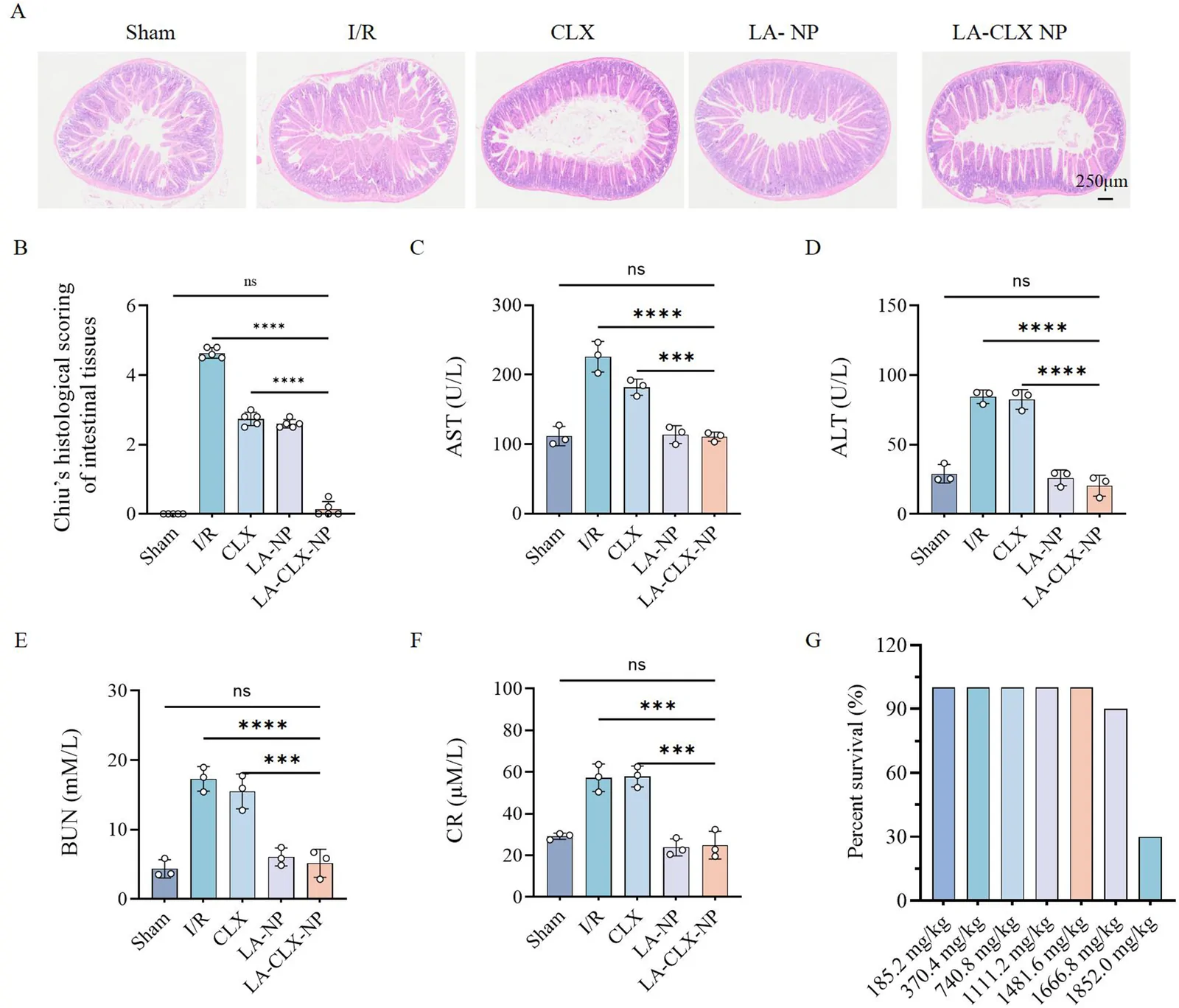
*In vivo* safety evaluation of LA-CLX-NP. **(A)** Representative H&E staining images of intestinal tissues from different treatment groups. Scale bar = 250 µm. **(B)** Chiu's histological scoring of intestinal tissues. **(C–F)** Serum biochemical parameters: **(C)** AST, **(D)** ALT, **(E)** BUN, **(F)** CR. **(G)** Percent survival of mice following a single intravenous injection of LA-CLX-NP at escalating doses (*n* = 10 per group). Survival was monitored daily for 14 days. Data are shown as mean ± SD (*n* = 3).*p < 0.05, **p < 0.01, ***p < 0.001 vs. I/R group.

Chiu's histological scoring (Figure 7B) corroborated these findings. The I/R group had a significantly elevated score compared with the Sham group. Treatment with free CLX or LA-NP partially reduced the scores, but both remained significantly higher than that of the Sham group. The LA-CLX-NP group showed the lowest score among all treatment groups, which was not significantly different from the Sham group, further confirming the protective effect of LA-CLX-NP on intestinal tissue.

To assess hepatic and renal function, we measured serum levels of aspartate aminotransferase (AST), alanine aminotransferase (ALT), blood urea nitrogen (BUN), and creatinine (CR) (Figures 7C–7F). The I/R and free CLX groups exhibited elevated AST and ALT levels compared with the Sham group, consistent with I/R-induced secondary liver injury and drug-associated hepatotoxicity. In contrast, the LA-CLX-NP group maintained AST, ALT, BUN, and CR levels comparable to those of the Sham group, with no significant differences observed. These results demonstrate that LA-CLX-NP does not induce detectable hepatotoxicity or nephrotoxicity at the therapeutic dose, supporting its safety for intravenous administration.

To further define the therapeutic index, we conducted an acute toxicity study via single intravenous injection using the up-and-down method. Seven dose groups were established (185.2, 370.4, 740.8, 1,111.2, 1,481.6, 1,666.8, and 1,852.0 mg/kg, corresponding to 1-, 2-, 4-, 6-, 8-, 9-, and 10-fold of the therapeutic dose), with 10 mice per group, and the animals were observed for 14 days.

The survival curve (Figure 7G and Supplementary Figure S7) showed that at doses≤1,111.2 mg/kg (6-fold the therapeutic dose), all animals survived with no observable signs of toxicity, and body weight gain was comparable to that of the control group. At 1,666.8 mg/kg (9-fold), 1 out of 10 animals died. At the highest dose of 1,852.0 mg/kg (10-fold), 7 out of 10 animals died during the observation period. Based on these results, the maximum tolerated dose (MTD) of LA-CLX-NP following a single intravenous injection in mice was determined to be 1,481.6 mg/kg, approximately 8-fold the therapeutic dose (185.2 mg/kg), indicating a favorable safety window for this nanoplatform.

Collectively, histopathological examination, serum biochemical analysis, and acute toxicity testing collectively demonstrate that LA-CLX-NP exhibits a favorable safety profile at the therapeutic dose (185.2 mg/kg), with no detectable hepatotoxicity, nephrotoxicity, or intestinal tissue damage. The MTD was determined to be at least 8-fold higher than the effective dose, indicating a relatively wide therapeutic index and supporting the translational potential of LA-CLX-NP for further preclinical safety evaluation.

## 4 Discussion

Postoperative pain management in patients with intestinal ischemia-reperfusion (I/R) injury faces an unresolved dilemma: effective analgesia often comes at the cost of delayed gastrointestinal recovery. Here, we present a reactive oxygen species (ROS)-responsive nanoplatform (LA-CLX-NP) that reconciles this contradiction by enabling on-demand delivery of the COX-2 inhibitor celecoxib specifically to the inflamed intestinal tissue. The system is built by conjugating DSPE-PEG with α-lipoic acid (LA), which serves dual roles as a ROS-labile “switch” and an intrinsic antioxidant. Upon encountering the high ROS microenvironment characteristic of I/R-injured gut, the disulfide bonds of LA are cleaved, triggering nanoparticle disassembly and rapid release of both celecoxib and LA. This design allows local accumulation of therapeutics, minimizing systemic exposure, thereby preserving gastrointestinal motility while achieving potent analgesia.

Our results demonstrate that LA-CLX-NP exhibits good colloidal stability and ROS-triggered release properties *in vitro*. In LPS-stimulated RAW 264.7 macrophages, the nanoparticles were efficiently internalized and suppressed COX-2 mRNA expression, reduced intracellular ROS, restored glutathione redox balance (increased T-GSH, decreased GSSG), normalized SOD activity, and rebalanced pro- and anti-inflammatory cytokines (decreased TNF-α/IL-6, increased IL-10). All these effects were superior to those of free celecoxib or LA alone. These synergistic anti-inflammatory and antioxidant effects were recapitulated in a mouse intestinal I/R model: LA-CLX-NP treatment significantly reduced diarrhea incidence, increased mechanical withdrawal threshold, decreased spontaneous abdominal licking and scratching behaviors, protected ZO-1 tight junction continuity, suppressed intestinal COX-2 overexpression, and normalized serum cytokine profiles. Collectively, LA-CLX-NP achieves effective pain relief while accelerating gastrointestinal functional recovery.

While the EPR effect has been widely exploited for passive targeting of nanocarriers to inflamed or tumor tissues, its clinical translation faces substantial challenges. It is well recognized that the EPR effect is often less pronounced in human patients than in rodent tumor or inflammation models, due to differences in vascular architecture, interstitial fluid pressure, and the heterogeneity of disease conditions. This discrepancy may limit the extent of nanoparticle accumulation in the injured intestine when translated to clinical settings. To address this limitation, future iterations of our platform could incorporate active targeting moieties—such as mannose for macrophage recognition or VCAM-1 antibodies for activated endothelium—to achieve lesion-specific accumulation independent of EPR heterogeneity. Additionally, strategies to actively modulate the gut microenvironment, such as temporary vascular permeability enhancement, may further improve delivery efficiency. These considerations have been incorporated into the discussion of our study's translational roadmap.

Comparison with recent findings in intestinal inflammation. Recent advances in nanomedicine for intestinal inflammatory diseases have highlighted the importance of ROS-responsive platforms for achieving site-specific therapy. A study by reported a redox-responsive nanoplatform that effectively alleviated colitis by scavenging ROS and repairing the intestinal barrier, demonstrating the feasibility of such approaches in inflammatory bowel disease models (28). In the context of intestinal I/R injury, a recent investigation demonstrated that targeted delivery of anti-inflammatory agents using ROS-responsive nanocarriers significantly attenuated tissue damage and improved functional recovery. Our LA-CLX-NP system aligns with these emerging strategies, yet offers distinct advantages: the dual functionality of LA as both a responsive linker and a therapeutic antioxidant enables carrier-payload synergy, while the co-delivery of celecoxib provides targeted COX-2 inhibition. Collectively, our findings extend the current understanding of ROS-responsive nanoplatforms from chronic intestinal inflammation to acute I/R injury, a clinically distinct but equally challenging scenario.

Although our study provides preliminary evidence for the short-term safety of LA-CLX-NP at the therapeutic dose, several critical aspects remain to be addressed prior to clinical translation. First, the long-term safety and potential chronic toxicity of the nanoparticle components—particularly the accumulation of DSPE-PEG or LA derivatives in reticuloendothelial organs—warrant systematic evaluation through repeated-dose toxicology studies. Second, given that celecoxib has been associated with cardiovascular risks (including hypertension and thromboembolic events) in long-term use, the cardiovascular safety profile of LA-CLX-NP requires careful assessment. The nanocarrier-mediated targeted delivery may reduce systemic celecoxib exposure and thereby mitigate such risks, but this hypothesis needs to be directly tested in appropriate animal models, including assessment of blood pressure, coagulation parameters, and vascular function. We have acknowledged these as critical next steps in the path toward clinical translation.

Nevertheless, the current system relies on passive (EPR-mediated) targeting. Future work could incorporate active targeting ligands (e.g., mannose for macrophages or VCAM-1 antibodies for activated endothelium) to further enhance lesion specificity. Moreover, the detailed molecular network linking LA-mediated ROS scavenging to NF-κB and Nrf2 pathways warrants deeper investigation using genetic or pharmacological tools.

## 5 Conclusion

In summary, this study provides a proof-of-concept for an intelligent, ROS-responsive analgesic strategy that harmonizes pain control with rapid gastrointestinal recovery. We have successfully constructed a LA-CLX-NP nanoplatform that exhibits ROS/pH dual-responsive drug release, efficient macrophage uptake, and synergistic anti-inflammatory and antioxidant effects. In a mouse intestinal I/R model, LA-CLX-NP significantly alleviated pain-related behaviors, protected intestinal barrier integrity, and promoted gastrointestinal functional recovery, with efficacy superior to that of free celecoxib or LA-NP alone. Mechanistically, these benefits are attributed to the suppression of COX-2/PGE2-mediated inflammatory pathways, preservation of ZO-1 tight junctions, and systemic rebalancing of pro- and anti-inflammatory cytokines. Although challenges such as EPR heterogeneity and long-term safety remain to be addressed, this nanoplatform offers a promising approach for postoperative management of intestinal I/R injury and holds potential for extension to other ROS-driven intestinal inflammatory conditions.

## Data Availability

The original contributions presented in the study are included in the article/Supplementary Material, further inquiries can be directed to the corresponding authors.
