# Supplementary material for: A ROS-responsive nanoplatform for synergistic pain relief and gastrointestinal recovery in intestinal ischemia-reperfusion injury
Source: Front Pediatr. 2026 Sep 21;14:1873783. doi: 10.3389/fped.2026.1873783 (PMC13635346; doi:10.3389/fped.2026.1873783)
Supplement: Supplementary file 1 [file Datasheet1.docx]

Support information

Table S1. Encapsulation efficiency and drug loading of LA-CLX-NP (n = 3).

| Parameter | Value |
| --- | --- |
| LA-NP fed | 5.00 mg |
| Celecoxib fed | 0.25 mg |
| Total nanoparticle weight (after lyophilization) | 4.82 ± 0.15 mg |
| Free celecoxib (in supernatant) | 0.115 ± 0.012 mg |
| Encapsulated celecoxib | 0.135 ± 0.012 mg |
| Encapsulation efficiency (EE%) | 54.0 ± 4.8% |
| Drug loading (DL%) | 2.80 ± 0.25% |

Table S2.Primer sequences for qPCR

| GAPDH | F: 5′-CCTTCCGTGTTCCTACCCC-3′ |
| --- | --- |
|  | R: 5′-CGCCTGCTTCACCACCTTC-3′ |
| COX-2 | F: 5′-GCATCCTGGGCTACACTGAG |
|  | R: 5′-TCCACCACCCTGTTGCTGTA |

Figure S1. ¹H NMR spectrum of CLX in DMSO.

Figure S2. ¹H NMR spectrum of LA in DMSO.

Figure S3. ¹H NMR spectrum of DSPE-PEG-LA in DMSO.

Figure S4. ¹H NMR spectrum of LA-CLX-NP in DMSO.

Figure S5. Representative immunofluorescence images of intracellular ROS levels in LPS-stimulated RAW 264.7 macrophages.(A) Representative fluorescence images of intracellular ROS levels detected by the DCFH-DA probe. Scale bar =10 μm. (B) Quantitative analysis of cellular ROS fluorescence intensity.Data are shown as mean ± SD (n = 3).*p < 0.05, **p < 0.01, ***p < 0.001 vs. I/R group.

Figure S6. Flow cytometric quantification of intracellular ROS levels in LPS-stimulated RAW 264.7 macrophages. (A) Representative flow cytometry histograms showing the fluorescence intensity distribution of DCFH-DA in each treatment group. (B) Quantitative analysis of mean fluorescence intensity (MFI).Data are shown as mean ± SD (n = 3).*p < 0.05, **p < 0.01, ***p < 0.001 vs. I/R group.

Figure S7. Survival curves of mice following single intravenous injection of LA-CLX-NP at various doses (n = 10 per group) over 14 days of observation.
